# Current status of the curricula of physiotherapy schools in Türkiye in terms of the usage of new rehabilitation technologies and measurement systems

**DOI:** 10.3389/fresc.2024.1504509

**Published:** 2024-12-20

**Authors:** Selda Uzun, Muhammed Yusuf Kahraman

**Affiliations:** ^1^ Sport Health Department, Faculty of Sport Sciences, Marmara University, İstanbul, Türkiye; ^2^ Sport Sciences and Athletes Health Research and Implementation Centre, Marmara University, İstanbul, Türkiye; ^3^ Sport Health Science PhD Program, Physical Education and Sport Department, Faculty of Sport Sciences, Marmara University, İstanbul, Türkiye; ^4^ Faculty of Sport Science, Haliç University, İstanbul, Türkiye

**Keywords:** physiotherapist, education, STEM, technology, academics

## History of physiotherapy in Türkiye

The foundation of the “Physiotherapy occupation” was laid in Türkiye with the establishment of Hacettepe University School of Physical Therapy and Rehabilitation (American school) in 1967 by Prof. Dr. İhsan Doğramacı as a four-year bachelor program.^1^ This institution was the only provider of physical therapy education in Türkiye for the following 25 years. In 1986, the Department of Physical Therapy and Rehabilitation was established at the Faculty of Medicine of İstanbul University under the chairmanship of Prof. Dr. Güzin DİLŞEN.^2^ Afterward, physical therapy and rehabilitation departments were established at Dokuz Eylül University (1993), Pamukkale University (1995), Abant İzzet Baysal University (1996), Dumlupınar University (1997), and Başkent University (1998), etc. (1). In 1983, a teamwork system carried out by specialist doctors, doctors in specialty training, nurses, physiotherapists, pharmacists, psychologists, and other professionals related to the branch was established in hospitals in order to improve the quality of services and to serve the patient in the best conditions.^3^ Despite all this, in the 90s, the public was not fully aware of what physiotherapy was for and doctors in other medical branches were not aware of the necessity of physiotherapy. In the 2000s, the profession of physiotherapy started to gain prominence in Türkiye due to the opening of special education centers for children with disabilities and the introduction of regulations requiring hospitals to have a sufficient number of physiotherapists based on patient volume. As a result, the demand for physiotherapists in Türkiye has steadily increased, leading to a rise in the number of physiotherapy departments in both state and foundation (private) universities across the country.

## Actual status of physiotherapy schools

Currently, there are physiotherapy schools in 56 universities [32 state (57.14%), 24 foundation/private (42.85%)] in Türkiye (85 million people), and approximately 6,000 physiotherapists have been graduating from these schools per year. However, the rapid increase in physiotherapy departments has led to the establishment of departments with insufficient academic competence, as some departments now have only three faculty members. The main objective of these physiotherapy departments is focused on graduating undergraduate students without considering MSc (2 years) and PhD (4 years) programs. There are 31 universities with MSc degrees and 15 offering PhD degrees in physiotherapy in Türkiye. There has been an increase of 35.91% in the number of students enrolled in physical therapy undergraduate programs in the last four years, as reported by the Council of Higher Education (Table 1).^4^ The number of full-time academic personnel with a physical therapy background has not reached a sufficient number with approximately 5 per faculty in a study conducted in 2014 (2). Despite the rapid expansion of physiotherapy programs and numerous postgraduate schools, the number of qualified academics has not increased correspondingly, possibly due to a lack of awareness or incentives for pursuing academic careers. Young physiotherapists are less interested in academic careers due to the long duration of the academic career (2 years master's degree and 4 years PhD), low scholarships and salaries, and the intense academic workload. Therefore, there should be better opportunities for newly graduated physiotherapists and support of academic career pathways in physiotherapy schools. For example, financial support can be provided by giving the opportunity to work in universities/hospitals for physiotherapists who are doing master's and doctorate, and thus academic careers can be incentivized.

**Table 1:** The number of students enrolled in physiotherapy departments of state and private universities in 2021, 2022, 2023, and 2024 in Türkiye.

	Years	2021	2022	2023	2024
**University**	**State**	3,369	3,700	3,818	4,119
	**Private**	958	1,726	2,063	3,222

## The curricula of physiotherapy schools

Physiotherapy includes all approaches to improve the functional abilities and motor capabilities of individuals throughout their lives. The primary aim of physiotherapy school is to teach future physiotherapists to perform a motor and functional assessment of patients with disability, define the main goals of the physiotherapist (PT) intervention, and deliver a PT treatment targeted to the motor signs responsible for functional limitations experienced by patients. The undergraduate physiotherapy program curricula are divided into three sub-levels: basic, internal, and surgical medicine in Türkiye (2). Additionally, medical science courses are integrated with physiotherapy and rehabilitation, covering orthopedic, neurological, neurochirurgic, pediatric, rheumatological, cardiopulmonary, gynecological diseases, and geriatrie, etc, (2). It is essential for the curriculum of physiotherapy schools to include courses from many different disciplines, because physiotherapists need to assess a patient from multiple perspectives and plan the correct or optimum treatment methods. The rapid development of technology in recent years has facilitated the preparation of both assessment and treatment procedures in physiotherapy. One of the programs developed to integrate rapidly developing technology into undergraduate curricula is the STEM (science, technology, engineering, and math) approach. STEM education is an interdisciplinary approach that covers all levels of education. Its mission is to enable students to approach problems from an interdisciplinary perspective and to acquire knowledge and skills through a new generation of educators (3, 4). It is unclear whether the STEM approach is integrated into traditional physiotherapy programs in Türkiye. The physiotherapy curriculum includes only physics as a quantitative course in the first year. However, mathematics courses are also needed for rapidly developing computer technologies, the use of new rehabilitation devices, and biosignal analysis. The lack of such courses makes it difficult for academics teaching in physiotherapy schools to update their course content within the scope of STEM. In addition, while the academic staff of sports sciences departments in Türkiye includes specialists in the fields of electronics, computer, physics, mechanical engineering, etc.,^5^ the academic staff in both undergraduate and MSc/PhD programs of Physiotherapy and Rehabilitation Departments are mostly physiotherapists and medical doctors (physical therapists, anatomists, orthopedists, etc.). In Türkiye, the academic staff of the Physiotherapy and Rehabilitation Departments are required to have a physiotherapy degree or to be medical doctors. The physiotherapy departments must have at least two faculty members from the fields of Physiotherapy or Medicine-Physical Therapy and Rehabilitation. The other faculty members can be from the fields of Occupational Therapy or Medicine (Physical Therapy and Rehabilitation, Orthopedics, Traumatology Neurology Brain and Neurosurgery Rheumatology Geriatrics Cardiology Chest Diseases Anatomy Physiology Sports Medicine).^6^ Thus, there is a lack of academic staff who can include or develop technology, engineering, and mathematics-based courses in the curriculum of physiotherapy education programs. Besides, there are only a few physiotherapists with master's and doctoral degrees in biomedical engineering in Türkiye. This situation limits the development of qualified physiotherapists who can apply innovative rehabilitation technologies and creates a gap in both education and practice. As a result, in order to increase the use of technology in physiotherapy; we believe that giving engineering experts the chance to work actively in physiotherapy schools will help to catch the latest technology in physiotherapy education.

Physiotherapists are very conservative about not allowing academics other than theirs and MDs to teach in physiotherapy schools. This is because of the idea that only physiotherapists should perform therapeutic exercises tailored to the patient's needs, technology-based interventions (e.g., virtual reality), neurological physiotherapy, manual therapy applications, etc. in order to protect patient health and professional ethics. Physiotherapists may be right to think this way to protect their professional ethics because, in order to acquire these complex therapy skills, it is necessary to have many years of experience after 4 years of undergraduate education or to participate in additional courses.

For the last decades, physiotherapists have been using state-of-the-art systems such as robotic devices (locomat, etc.), wearable technologies, and virtual reality, etc. in physiotherapy hospitals and universities in Türkiye as in the whole world. Similarly, physiotherapists employed in the academic staff of universities and research hospitals carry out many research projects using the systems [force platform, surface EMG (sEMG), motion capture systems, and isokinetic, etc.]. However, even if physiotherapists know how to use these systems, they encounter challenges in terms of working mechanisms, raw data, analysis, and interpretation of data. Therefore, they collaborate with engineering academicians working in departments other than physiotherapy departments in their academic research. Physiotherapists who do not have the opportunity to collaborate with engineers usually prefer to use systems with analysis programs provided by the manufacturer, and the salesmen already provide the required training for the use of the entire system at the time of sale. However, few operators are informed in this way and if they are temporarily or permanently replaced this knowledge is lost and the equipment may be abandoned or misused. In addition, this type of training by the salesman may be biased.

In recent years, congresses and symposia have been organized on the use of technology in rehabilitation under the leadership of Turkish physiotherapists. It is not mandatory to participate in these meetings for physical therapists or students. In these meetings, the heads of physiotherapy departments invite academicians or experts from different engineering departments to discuss topics such as robotic rehabilitation, artificial intelligence, wearable technologies, virtual reality, and telerehabilitation. However, there is almost no participation from physiotherapists in Türkiye except for a few people in the meetings organized by the ISEK (The International Society of Electrophysiology & Kinesiology)^7^ community, which combines the disciplines of electrophysiology and kinesiology established to study human movement and the neuromuscular system. These participants are usually physiotherapists who have master's or doctorate degrees in biomedical engineering departments in Türkiye. However, JEK (Journal of Electromyography and Kinesiology), the official journal of ISEK, attracts more interest from Turkish physiotherapists and other scientists, and a few publications are accepted every year. In the meantime, we think that the awareness of undergraduate or postgraduate students about ISEK or JEK is limited to their specific interests. In order to transfer this knowledge to the younger generation, we think that the STEM approach in physiotherapy education should be integrated into all physiotherapy education programs. Thus, physical therapists can receive multidisciplinary and technology-based education, which enables them to utilize sophisticated devices effectively. In this way, PTs and occupational therapists who are proficient in analyzing and interpreting data will have the opportunity to implement appropriate rehabilitation strategies in their clinical practice. Furthermore, the establishment of rehabilitation engineering institutes in Türkiye, in addition to biomedical engineering departments, could facilitate collaboration between physiotherapists and engineers.

Looking at the undergraduate education of physiotherapy schools in Türkiye, only a few universities have a single course called “Use of the Latest Technologies in Rehabilitation”. The course covers topics such as the functions and uses of assistive devices, including inertial sensors, wearable robotics, muscle stimulation techniques, and improving physical activity technologies used in the rehabilitation process. On the other hand, as the first university in Türkiye to establish a physiotherapy department, Hacettepe University offers a course called “Technology-Based Assessment in Physiotherapy”, which covers topics such as isokinetic strength, foot pressure, posture, scapular kinematics and assessment of muscle activation levels.^8^ However, the content of these courses is not comprehensive and includes only general information about new technology equipment. It only covers superficial topics such as the benefits and difficulties of technology and digitalization. The reason why the content of these technology-based courses is so superficial is that academics with engineering backgrounds are not assigned to physiotherapy departments. These courses should include detailed analysis and interpretation of the data obtained from these measurement systems, focusing not only on the operational benefits and how to use them but also on developing practical skills in data processing, clinical application, and integration of technology.

There is generally no standardization between the curricula of physiotherapy and rehabilitation departments. Most of the theoretical and practical courses are similar. Furthermore, the existing courses on electrophysiological research methods are elective and have limited sEMG testing and training materials for analysis in university curricula. Additionally, there are no engineering-based academics commissioned at each level of physiotherapy programs. In the curriculum of physiotherapy schools, literature discussion, and article assignments are included to develop occupational content, process analysis, and scientific literacy. However, there are some deficiencies in the curriculum in terms of creative and innovative thinking, design, entrepreneurship, and technological literacy. These deficiencies may be compensated by some organizations organized by some student groups according to their interests (journal clubs, etc.) and scientific project competitions organized by TÜBİTAK (The Scientific and Technological Research Council of Türkiye), drawing students’ attention to research on entrepreneurship and technology. The occupational courses offered in the departments include only theoretical and practical applications of therapy methods used in basic, internal, and surgical medicine. Project-based and problem-based learning methods within the STEM approach are not applied in these courses where just therapy methods are explained. “Kinesiology”, “Biomechanics”, and “Measurement and Evaluation in Physiotherapy” courses include a mechanical approach to musculoskeletal systems and include posture, joint motion, anthropometrics, and muscle strength assessment. In the undergraduate and graduate programs of only two institutions (Hacettepe and Süleyman Demirel Universities), muscle activation assessments are covered in one course in one semester. The reason for this deficiency can be attributed to the absence of academicians with engineering backgrounds who are experts in sEMG and the lack of sEMG training materials in the course content. Due to this lack, there are a few specialists in muscle activation testing and analysis. In addition, the lack of expertise in testing, analysis, and interpretation to use sEMG systems precludes the use of these devices, even though physical rehabilitation devices (EMS, ultrasound, etc.) are used in clinics.

In some of the course curricula in physiotherapy schools in Türkiye, information about neuromuscular electrophysiology and bioelectric signals (ECG, EEG) is generally taught theoretically.^9^ In some schools, there is the use of ECG in the clinic, but there is not enough education to gain competence in academic research on signaling. The use of EEG devices in diagnosis (epilepsy etc.) is mentioned in neurophysiology/electrophysiology etc. courses. EEG-based neurorehabilitation applications are applied in a few private physical therapy clinics (by MDs), and the use or analysis of EEG is of little interest to physiotherapists, even those doing academic research. However, research on EEG/ECG and sports performance is of more interest to sports scientists working in collaboration with engineers in the field of sports sciences.

## Medical devices used in physiotherapy

According to the regulation of health professionals, “The physiotherapist performs the necessary practices to improve the movement and/or eliminate physical dysfunctions of the patients depending on the diagnosis and direction of the physical therapy and rehabilitation doctors for treatment. Additionally, the physiotherapist determines the appropriate dimensions and specifications for the use of rehabilitation devices and technologies”. The devices and technologies are used to support exercise applications and indications of specific complications in patients. In clinics where physical therapy and rehabilitation doctors and physiotherapists work together, devices such as EMS, Tens, and Ultrasound are generally used to reduce pain and enhance healing, and devices such as Goniometer, Inclinometer, and Handheld Dynamometer are used for the indication of physical dysfunctions in patients. However, there is a lack of use of sEMG systems showing muscle activity parameters in rehabilitation clinics. Thus, it may limit the opportunity to use sEMG to answer to specific biomechanical queries or relevant clinical questions representing the rationale on which the physiotherapy treatment is based on.

## Providing of research grants

The main foundation that provides research grants is the Scientific and Technological Research Council of Türkiye (TÜBİTAK).^10^ The Council supports researchers in various fields, such as health and engineering, with periodic research calls. The main grant which is provided by TÜBİTAK is “1001 The Scientific and Technological Research Projects Funding Program” for scientific research and the requested budget from TÜBİTAK can be a maximum of 50.000 euro [including Scholarship, excluding Project Incentive Bonus (PTI) and overhead] without annual budget limit in this program. The other grants such as 1001, 1002, 1003, 3501, and 3001 are short-term national support programs for scientific research ideas. The funding provided by the national council might also be used for the training of physical therapists and movement specialists and for preparing new training materials or upgrading the existing professional figures.

## Artificial intelligence (AI) in physiotherapy

The use of artificial intelligence-based systems in clinical settings is rapidly increasing in healthcare. Examples of the use of these systems in clinical practice include video analysis, natural language processing (NLP), robotics (myoelectric control with symbiotic neuroprostheses), brain-computer interface technology, perioperative medicine, personalized healthcare, expert systems and predictive algorithms to classify data and make recommendations, etc. (5–7). In this context, it is suggested that some of the routine physical and cognitive tasks in physical therapy practice may be automated by AI-based systems in the near future. If successful clinical practice in the future requires understanding how to interpret the recommendations of machines, when to give them manual control, and when to ignore them, physical therapy education will need to adapt accordingly (6, 7). Therefore, the advantages and disadvantages of using AI in clinical practice should be included in the curriculum to provide an overview of developments related to AI in physical therapy.

## Future perspective

Finally, our future perspective for the physical therapy field is that educational materials involving rehabilitation technologies such as sEMG, related to STEM must be added to the curricula of physiotherapy programs. In this regard, it was suggested that the fast development of technology requires new competencies by the next generation of physical and occupational therapists in the fields of science, technology, engineering, and mathematics (STEM), which, in turn, requires a new educational curriculum (8). Rehabilitation clinics often cannot use sEMG, motion capture systems, etc. in musculoskeletal system evaluations due to their high cost and limited knowledge of physical therapists and movement scientists. If existing educational materials (9–14) are integrated into undergraduate education programs, clinical applications including sEMG tests and data interpretation might pave the way.

In Türkiye, biomedical engineers in many universities apply technological measurements in the field of rehabilitation at an academic level (professor, PhD). Biomedical engineers working as academicians generally look favorably on joint research with physicians (neurologists, physical therapists, and orthopedics) and physiotherapists. The transfer of this information to hospitals and private clinics has recently become important. However, physiotherapists prefer to learn how to use automated systems rather than increase their competence in STEM (biosignal analysis). We have two different suggestions on this subject (1) technological measurements in rehabilitation should be performed by physiotherapists because it would be ethically problematic for engineers or clinical technologists who do not know human body biomechanics and kinesiology to interpret the results and provide recommendations to the patients, or (2) physiotherapists should collaborate with biomedical or other engineers for technological measurements and analyses. To achieve all of these, the STEM approach should be integrated into the curriculum of physiotherapy schools and engineers should be allowed to work in physiotherapy departments.

## Footnotes

^1^Retrived November 10, 2024, from https://ftrfakulte.hacettepe.edu.tr/en

^2^Retrived November 10, 2024, from https://fizyoterapisabif.iuc.edu.tr/tr/content/bolumumuz/tarihce

^3^Retrived November 10, 2024, from https://sakur.uludag.edu.tr/files/17.pdf

^4^Retrived November 10, 2024, from https://yokatlas.yok.gov.tr/netler-tablo.php?b=11016

^5^Retrieved November 10, 2024, from https://sporbilimleri.hacettepe.edu.tr/index.php?lang=en&pid=1764

^6^Retrieved November 10, 2024, from https://www.yok.gov.tr/Documents/Kurumsal/egitim_ogretim_dairesi/Yok-tarafindan-Asgari-Kosullari-Belirlenen-Programlar/saglik_Programlari.pdf

^7^Retrived November 10, 2024, from https://isek.org/

^8^Retrieved November 10, 2024, from https://bilsis.hacettepe.edu.tr/oibs/bologna/index.aspx?lang=tr&curOp=showPac&curUnit=761&curSunit=762#

^9^Retrieved November 10, 2024, from https://ftr.deu.edu.tr/fizyoterapi-ve-rehabilitasyon-2024-2025-ogretim-plani/

^10^Retrived November 10, 2024, from https://tubitak.gov.tr/en
